# Assembly and annotation of the ‘Golden Delicious’ Doubled-Haploid GDDH18 apple genome

**DOI:** 10.1093/g3journal/jkag104

**Published:** 2026-04-27

**Authors:** Marine Salson, Christelle Lalanne, Maryline Cournol, Sylvain Hanteville, Damien Hinsinger, Patricia Faivre-Rampant, Aurélie Bérard, Arnaud Bellec, Stéphane Cauet, Nathalie Choisne, Sébastien Aubourg, Anne-Laure Fanciullino, Jean-Marc Celton, Sandrine Balzergue

**Affiliations:** Université Angers, Institut Agro, INRAE, IRHS, SFR QUASAV, Angers F-49000, Pays de la Loire, France; Université Angers, Institut Agro, INRAE, IRHS, SFR QUASAV, Angers F-49000, Pays de la Loire, France; Université Angers, Institut Agro, INRAE, IRHS, SFR QUASAV, Angers F-49000, Pays de la Loire, France; Université Angers, Institut Agro, INRAE, IRHS, SFR QUASAV, Angers F-49000, Pays de la Loire, France; Université Paris-Saclay, Centre INRAE Île-de-France Versailles-Saclay, EPGV, Evry 91057, Ile-de-France, France; Université Paris-Saclay, Centre INRAE Île-de-France Versailles-Saclay, EPGV, Evry 91057, Ile-de-France, France; Université Paris-Saclay, Centre INRAE Île-de-France Versailles-Saclay, EPGV, Evry 91057, Ile-de-France, France; INRAE, CNRGV French Plant Genomic Resource Center, Castanet Tolosan F-31320, Occitanie, France; INRAE, CNRGV French Plant Genomic Resource Center, Castanet Tolosan F-31320, Occitanie, France; Université Paris-Saclay, INRAE, AgroParisTech, Institute Jean-Pierre Bourgin for Plant Sciences, Versailles 78000, France; Université Angers, Institut Agro, INRAE, IRHS, SFR QUASAV, Angers F-49000, Pays de la Loire, France; Université Angers, Institut Agro, INRAE, IRHS, SFR QUASAV, Angers F-49000, Pays de la Loire, France; Université Angers, Institut Agro, INRAE, IRHS, SFR QUASAV, Angers F-49000, Pays de la Loire, France; Université Angers, Institut Agro, INRAE, IRHS, SFR QUASAV, Angers F-49000, Pays de la Loire, France

**Keywords:** genome assembly, *Malus domestica*, de novo gene annotation, transposable element annotation

## Abstract

Apple (*Malus domestica* Borkh.) is an important fruit crop cultivated worldwide in temperate regions. Due to their frequent high content in repetitive DNA sequences and large size, plant genomes have long been considered challenging to assemble. A first reference genome of a “Golden Delicious” doubled-haploid apple tree, GDDH13, was previously produced in 2017. The chromosomes of GDDH13 present however a high percentage of N stretches, around 12%, and above 8% of the assembly was not anchored on chromosomes. Here, we provide a chromosome-level assembly of another “Golden Delicious” doubled-haploid apple tree, GDDH18. Interestingly, this tree presents phenotypic differences with GDDH13, notably associated with fruit size. In this assembly, 99.4% of the sequences were assembled into 17 contigs corresponding to the 17 expected chromosomes of apple, and all but 1 of these 17 contigs present telomeric repeats at both their extremities. A high complete benchmarking universal single-copy ortholog (BUSCO) score and a high LTR assembly index of 99.5% and above 22, respectively, attest to the high quality of the assembly and to the completeness of both the gene and the repetitive space. A de novo annotation of the assembly was produced, allowing the prediction of 51,892 protein-coding genes, of which 93% are functionally annotated. A total of 6,239 additional genes were functionally annotated in comparison with the GDDH13 assembly. This high-quality assembly of GDDH18 along with a de novo annotation will help to better understand the phenotypic differences between the trees GDDH13 and GDDH18 and will serve as an enhanced reference genome for advancing the study of apple genomics.

## Introduction

Apple (*Malus domestica* Borkh.) belongs to the Rosaceae family and is an important fruit crop cultivated worldwide in temperate regions. It has a diploid genome of around 650 Mb with 17 chromosomes ranging from 31 to 59 Mb (2*n* = 2*x* = 34) and is composed of approximately 57% of transposable element (TE) repeats (Daccord et al. 2017). A Rosaceae ancestor from which the apple originates notably underwent a whole genome duplication (WGD) around 27  Mya (Lallemand et al. 2023). The domestication of apple likely began in the Tian Shan Mountains in Central Asia between 4,000 and 10,000 years ago, and the wild Central Asian species *Malus sieversii* has been shown to be the main contributor to the genome of the domesticated apple *M. domestica* (Harris et al. 2002; Velasco et al. 2010). The spread of domesticated apples to Europe through the Silk Road was associated with additional contributions of other local wild species, including *Malus sylvestris* and *Malus orientalis* (Cornille et al. 2014, 2019). Hybridization with wild relatives thus played an important role in the evolution of the cultivated apples (Cornille et al. 2012, 2014; Duan et al. 2017; Sun et al. 2020). A doubled-haploid and highly homozygous “Golden Delicious” line called Golden Delicious doubled-haploid 13 (GDDH13) was developed thanks to breeding effort (Lespinasse et al. 1998; Daccord et al. 2017). This highly homozygous line allowed to facilitate the assembly of a reference genome (Daccord et al. 2017).

Plant genomes have long been considered challenging to assemble, notably because they are often characterized by high repetitiveness, large genome size, or high ploidy levels (Belser et al. 2018; Kong et al. 2023). A number of projects have however permitted the production of several high-quality plant genomes these last few years, notably thanks to the development and improvement of long-read sequencing technologies (Belser et al. 2018, 2021; Istace et al. 2021; Aury et al. 2022; Salson et al. 2023). A chromosome-level assembly of GDDH13 was produced in 2017 (Daccord et al. 2017). However, this genome contains 12% of ambiguous bases (N), telomeric repeats are missing at the ends of most of the chromosomes, and 52.7 Mb of unanchored contigs were grouped onto an artificial pseudochromosome 0.

Here, we provide a high-quality assembly of the apple line Golden Delicious doubled-haploid 18 (GDDH18). This tree originates from the same haploid seedling used to generate GDDH13 (Daccord et al. 2017) but differs by producing significantly smaller fruits and a higher number of seeds, which facilitates laboratory experimentation. This GDDH18 assembly showed improved continuity and better quality metrics in comparison with the previous GDDH13 assembly (Daccord et al. 2017). Additionally, we also provided a de novo annotation of this GDDH18 assembly. A high-quality assembly of GDDH18, combined with a de novo annotation, will not only facilitate the identification of genes or alleles associated with fruit development and clarify the phenotypic differences between GDDH13 and GDDH18, but more importantly, it will provide an enhanced reference genome. This improved genomic resource represents a cornerstone for advancing the study and understanding of apple genomics, enabling more precise investigations and fostering future applications in fruit biology and breeding.

## Materials and methods

### Plant materials and sequencing

Young leaves of a “Golden Delicious” doubled-haploid tree GDDH18 (Daccord et al. 2017) from UE Horti orchard (https://doi.org/10.15454/1.5573931618268674E12) were sampled. High DNA molecular weight was extracted with the NucleoBond HMW DNA kit (Macherey-Nagel, Düren, Germany) according to the manufacturer’s instructions. DNA quality was assessed with FemtoPulse (Agilent, CA, USA) after SRE-XL kit purification (Pacific Biosciences, Menlo Park, CA, USA) to eliminate fragments smaller than 30 Kb, and quantity was checked with a Qubit 4 fluorometer (Thermo Fisher Scientific, MA, USA).

A sequencing library was built with the ligation Oxford Nanopore sequencing kit V14 (SQK-LSK114) using 1,000 ng as input, with a ligation time of 30 mn and using the LFB buffer. Sequencing was performed by using a R10.4.1 flowcell on a PromethION 24 instrument for 96 h.

After the first 24 h, the flowcell was washed with a nuclease-flush according to the manufacturer's instructions and then reloaded with a fresh library built as above.

Raw signal was live-base called with Dorado v7.8.3+f64462b6f and the SUP model (super accurate), as implemented in minKNOW v6.4.8. Taxonomic affiliation of the basecalled reads was assessed with centrifuge 1.0.3 (Kim et al. 2016) to detect contamination during the library and sequencing processes.

### Long-read assembly

Long reads were filtered using Filtlong (v. 0.2.0, --length_weight 10 https://github.com/rrwick/Filtlong). Several read coverages were assessed for the assembly (ranging from 30× to 60×, Supplementary Table 1). The 2 assemblers Flye (v. 2.9.2, Kolmogorov et al. 2019) and Hifiasm (v. 0.25.0, Cheng et al. 2021, 2022, 2024) were tested. Telomeres were sought on the different assemblies using quarTeT (v. 1.2.5, Lin et al. 2023). We selected the assembly with the minimum number of contigs and for which telomeres were found at both extremities for a maximum number of chromosomes. We used both FCS-adaptor and FCS-GX (Astashyn et al. 2024; https://github.com/ncbi/fcs) to detect and remove adaptors and contaminations from foreign organisms in the selected assembly. We used publicly available paired-end short reads of the GDDH18 tree (run SRR5351715, BioProject PRJNA379390) for polishing of the assembly. The short reads were aligned to the assembly using bwa-mem2 (v.2.2.1, mem option, Li and Durbin 2010; Vasimuddin et al. 2019), and we only kept properly paired reads using samtools (v. 1.9, -f 0x02 option, Danecek et al. 2021). Hapo-G was then used to polish the assembly using paired-end short reads (v. 2.31, default parameters, Aury and Istace 2021).

### Quality assessment and comparison with previous *Malus* genomes

The completeness of the gene space assembly was assessed using benchmarking universal single-copy orthologs (BUSCOs) (v. 6.0.0, Manni et al. 2021) and 3 databases: embryophyta_odb12 (2,026 BUSCO genes), viridiplantae_odb12 (822 BUSCO genes), and rosaceae_odb12 (10,071 BUSCO genes). We computed the LTR assembly index (LAI) score (Ou and Jiang 2018; Ou et al. 2018) using LTR_retriever (v 3.0.4) to assess the continuity of the assembly based on the repeat space following the protocol described here: https://github.com/oushujun/LTR_retriever. We used LTRharvest and LTR_FINDER_parallel to identify LTR. LTR_retriever was then used to compute the LAI score. In order to assess the impact of the polishing step on the short reads, we computed both the LAI and the BUSCO scores of the GDDH18 assembly before and after the polishing step.

For comparison purposes, the BUSCO and the LAI scores were also computed for the GDDH13 assembly (Daccord et al. 2017) and a recent high-quality “Golden Delicious” diploid phased genome, GDT2T (Su et al. 2024). We used the same databases and the same procedures as previously described to compute the BUSCO and the LAI scores.

We used D-GENIES to compare the chromosomes of the GDDH18 assembly with the chromosomes of both GDDH13 and GDT2T (v. 1.4, hide noise option enabled and small matches filtered, Cabanettes and Klopp 2018).

### Telomeric and centromeric repeat identification

We used quarTeT (v. 1.2.5, default parameters, Lin et al. 2023) to identify telomeric repeats on the chromosomes of the 3 “Golden Delicious” assemblies GDDH18, GDDH13, and GDT2T.

We mapped the 9,716 bp HODOR (High cOpy golDen deliciOus Repeat, accession KX869746, Daccord et al. 2017) sequence to the GDDH18 chromosomes using Minimap2 (Li 2018, -N 1000 -p 0.5 parameters) and computed the number of HODOR alignments found on nonoverlapping genomic windows of 1 Mb for each chromosome. We only kept alignments longer than 3,000 bp. We used these alignments to estimate the putative centromere position on the GDDH18 assembly. We performed the same analysis on the GDDH13 genome for comparison.

### Mitochondrial and chloroplast sequence identification

We used Oatk (Zhou et al. 2025, v.1.0, default parameters) with the angiosperm database for assembling the mitochondrial and chloroplastic genomes using all the Nanopore long reads. We also mapped the mitochondrion (NCBI accession NC_018554.1, Goremykin et al. 2012) and the chloroplast (NCBI accession NC_061549.1, Li et al. 2022) sequences to the GDDH18 assembly obtained with Hifiasm, using Minimap2 (v. 2.22, default parameters, Li 2018). This allowed to identify the contigs corresponding to mitochondrial and chloroplast sequences and remove them from the final assembly.

### Gene annotation

Structural annotation of the *M. domestica* GDDH18 genotype was performed using the EuGene pipeline (Sallet et al. 2019; Carrere and Gouzy 2023). This integrative gene prediction workflow combined multiple sources of evidence, including transcript alignments and protein homology searches. Transcriptome assemblies (Supplementary Table 2) were used to guide gene model prediction, while protein sequence homology was assessed against several reference databases, including Swiss-Prot and the UniProt/TrEMBL plant subset. Detection and masking of transposable elements (TEs) were carried out using the Dfam consensus sequence database (https://dfam.org) and the TransposonPSI protein database (http://transposonpsi.sourceforge.net).

Functional annotation of predicted protein-coding genes was performed using a combination of eggNOG-mapper v2.1.12 (Cantalapiedra et al. 2021), BLAST + v2.15.0 (Altschul et al. 1990), InterProScan v5.64-96.0 (Jones et al. 2014), and KofamScan v1.3.0 (Aramaki et al. 2020). Outputs from these tools were integrated to produce final annotation files in GFF3 and TSV formats, prioritizing eggNOG annotations when overlaps occurred between sources. We applied the default parameter values for all analyses, unless otherwise specified.

We estimated the proportions of the genome in protein-coding genes using the outputs of this pipeline.

### Identification of syntenic blocks

Identification of syntenic genes along the chromosomes of the GDDH18 assembly was performed using i-ADHoRe (Simillion et al. 2008; Proost et al. 2012) and an approach previously used for GDDH13 (Lallemand et al. 2023). For the identification of homologous genes, we first used blastP (e-value 10-5, max target seq 5, Camacho et al. 2009) and aligned all the proteins identified in GDDH18 vs all. The resulting homology information was then used as input with i-ADHoRe (v.3.0, Proost et al. 2012) for the construction of syntenic blocks. We used the same parameters for i-ADHoRe (v.3.0, Proost et al. 2012) as previously used for GDDH13 (Lallemand et al. 2023): cluster type = colinear; tandem gap = 15; gap size = 30; cluster gap = 30; *q* value = 0.75; prob cutoff = 0.01, and anchor points = 5. The identification of homologous genes between the chromosomes allowed the reconstruction of syntenic fragments that were displayed using Circos (Krzywinski et al. 2009). To estimate the number of ohnologous genes, we retained the pairs from the last WGD 27 Mya (Lallemand et al. 2023) belonging to the ohnologous chromosome pairs: 01 to 07, 01 to 15, 02 to 07, 02 to 15, 03 to 11, 04 to 06, 04 to 12, 05 to 10, 06 to 14, 08 to 15, 09 to 17, 12 to 14, and 13 to 16.

### TE annotation

The TEannot pipeline from the REPET package V3.0 (Quesneville et al. 2005; Flutre et al. 2011; Hoede et al. 2014; https://urgi.versailles.inrae.fr/Tools/REPET/TEannot-tuto) was used with default parameters to annotate TE copies in the GDDH18 genome. We used the consensus library of TE previously constructed for GDDH13 (Daccord et al. 2017; https://urgi.versailles.inrae.fr/repetdb/report.do?id=49000001), to identify repetitive elements in the GDDH18 assembly. A total of 2,456 TE consensus sequences were used, ranging from 358 to 17,023 bp, with a mean size of 3,150 bp. We estimated the percentage of the different classes of TE copies in the GDDH18 genome, using the GFF3 file generated with TEannot.

## Results and discussion

### Long-read sequencing

We generated 4.4 M reads “PASS” with a quality score *Q* larger than 10 (85.5% of the total sequencing data) with a N50 of 31.8 kb and a mean *Q* value of 20.48, totaling 82.45 Gb of sequencing data. Of these reads, 91.64% were complete with Nanopore adapters kept at both ends, indicative both of the quality of the sequencing and of the library. A taxonomical analysis of contaminants was performed with centrifuge1.0.3, using a 10,000 reads subsample (with options -k 15 --min-hitlen 26 --host-taxids 3750 --exclude-taxids 32644,28384,12908,77133). This analysis showed no significant contamination, with only 0.30% and 0.08% of bacterial and fungal reads, respectively (Supplementary Tables 3 to 5).

### Assembly of the long reads

We tested long-read assembly with both Hifiasm (Cheng et al. 2021, 2022, 2024) and Flye (Kolmogorov et al. 2019) and evaluated different depths ranging from 30× to 60× (Supplementary Table 1). A depth of 45× yielded the least fragmented assembly using Hifiasm, composed of a maximum number of contigs with telomeric repeats at both extremities (Supplementary Table 1). This assembly was obtained using 508,796 filtered reads, with a N50 and a mean length of 57 kb (Supplementary Table 6). A total of 26 contigs were obtained with a N50 of 36 Mb (Table 1). The N50 of the contigs of the GDDH18 assembly was 58-fold larger than GDDH13 (Supplementary Table 7). The total size of the assembly was 658 Mb (Table 1), corresponding to the expected size of the apple “Golden Delicious” haploid genome (Daccord et al. 2017; Su et al. 2024).

**Table 1.** Assembly statistics.

Total assembly	
Total length	658,096,990 bp
GC content	38.9%
Complete BUSCOs viridiplantae_odb12 (*n* = 822)	99.5%
Complete and duplicated BUSCOs	55.0%
Fragmented BUSCOs	0.1%
Missing BUSCOs	0.4%
**Chromosomes**	
Number of chromosomes	17
Total length of chromosomes	655 Mb
Percentage of Ns	No gap
**Contigs**	
Number of contigs	28
Longest contig	59 Mb
N50 (contigs)	36 Mb
**Gene annotation**	
Total number of genes	65,170
Number of protein-coding genes	51,182
Number of protein-coding and functionally annotated genes	48,379

### Identification of telomeric repeats and of the chromosomes

**Fig. 1.**
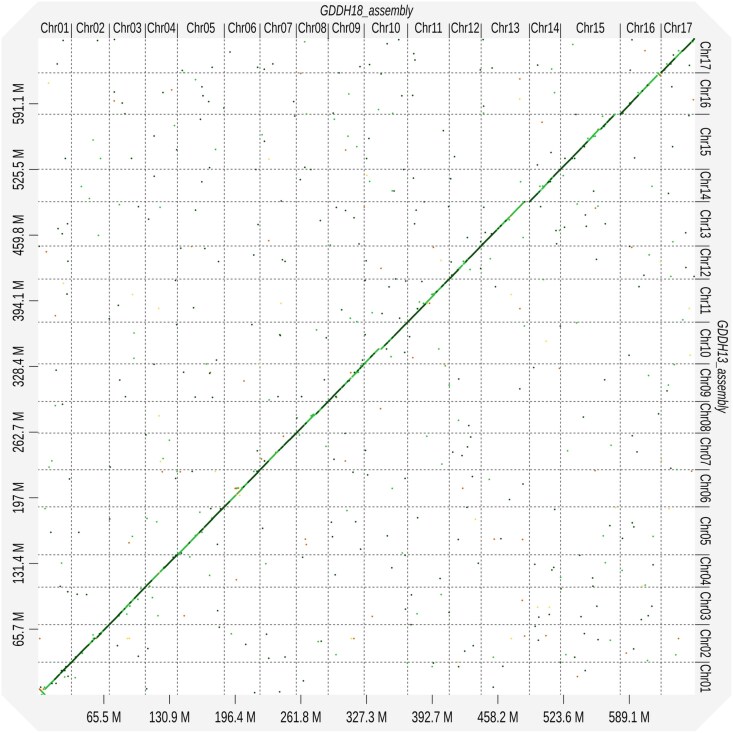
Chromosome-to-chromosome alignments between GDDH13 and GDDH18. The 17 longest contigs of the GDDH18 assembly identified as the 17 putative chromosomes of the apple genome are aligned to the 17 chromosomes of GDDH13 (Daccord et al. 2017). On the *x* axis are the GDDH18 chromosomes, and on the *y* axis are the GDDH13 chromosomes.

Telomeric repeats were identified at both extremities of 16 contigs, likely corresponding to 16 chromosomes (Supplementary Fig. 1 and Supplementary Table 8). In comparison, no chromosome of the GDDH13 assembly (Daccord et al. 2017) showed telomeric repeats at both ends (Supplementary Table 7). Telomeric repeats were also found at 1 single extremity of 2 other contigs (Supplementary Fig. 4). One of these contigs, ptg00006l, had a size of around 47 Mb, close to the expected size of chromosome 5 of GDDH13 (Supplementary Table 8). The other smallest contig was 498 kb in size (ptg000023l, Supplementary Table 8). While this telomeric region might represent 1 extremity of chromosome 5, the contig mapped ambiguously to multiple positions of the GDDH13 and the GDT2T genomes. It was thus included in the Chr00 (see the corresponding section). Alignment of the 17 largest contigs of the GDDH18 assembly to the GDDH13 chromosomes confirmed that these contigs corresponded to the 17 chromosomes of the apple genome (Fig. 1). The remaining 9 small contigs that were not included in the nuclear genome assembly represented 0.6% of the assembled sequences, highlighting the high contiguity and completeness of the contig sequences.

### Identification of mitochondrial and chloroplast sequences

The 9 contigs shorter than 1 Mb in size were assembled separately and were not integrated into the putative chromosomes by Hifiasm. Three contigs (ptg000024l, ptg000021c, and ptg000022c) were mapped to the published mitochondria and chloroplast genomes of apple (NCBI accessions NC_018554.1, Goremykin et al. 2012, and NC_061549.1, respectively, Li et al. 2022) with sequence identities between 75% and 100% (Supplementary Fig. 2). The contig ptg000022c notably seemed to correspond to multiple adjacent chloroplast sequences, based on visualization of the alignments (Supplementary Fig. 2). These contigs that aligned to the mitochondria and the chloroplast genomes were discarded from the final assembly. OatK (Zhou et al. 2025) allowed the assembly of a 162 kb sequence that aligned all along a previously published chloroplast apple sequence (NC_061549.1 NCBI accession, Li et al. 2022) with high identity (Supplementary Fig. 3, Supplementary Table 9 for annotation). However, no sequence was assembled with OatK for the mitochondrial genome. A previous study showed that the apple mitochondrial genome displays little similarity with other angiosperm mitochondrial genomes (Goremykin et al. 2012), which could make it difficult to reconstruct the sequences (Ni et al. 2025), notably when using databases.

### Construction of a Chr00 and of the final assembly

We constructed a Chr00 (named MdG1800) totaling 2.2 Mb and comprising 3 contigs (ptg000013l, ptg000019l, and ptg000023l of 587, 782, and 498 kb, respectively), composed of repetitive sequences (identified by FCS-GX, Supplementary Table 10), and 2 additional contigs (ptg000025l and ptg000026l of 253 and 97 kb, respectively). The Chr00 gathers the sequences that have not been anchored with enough confidence to a chromosome. The Chr00 of the GDDH18 assembly was 24-fold smaller than the Chr00 of the GDDH13 assembly (Daccord et al. 2017), demonstrating a greater completeness of the assembly of the chromosomes (Supplementary Table 7).

Using FCS-GX, 1 contig, ptg000020c, was found to be similar to a *Cannabis sativa* sequence (Supplementary Table 10) and was thus removed from the assembly. The final assembly is composed of 17 chromosomes (MdG1801-MdG1817, Fig. 1, Supplementary Table 10), including 16 chromosomes with telomeric repeats at both ends (Supplementary Table 8, Supplementary Fig. 1), along with the Chr00, the mitochondrial, and the chloroplast sequences. Three short adaptor sequences were found and removed from the final assembly (Supplementary Table 11).

### Quality metrics

The complete BUSCO scores obtained for the whole genome assembly of GDDH18 were high, between 97.4% and 99.8% depending on the database used (Table 1 and Supplementary Table 7), and were indicative of the high completeness of the gene assembly. These BUSCO scores were notably slightly higher than those found on GDDH13, between 96.0% and 98.1% using the same databases (Supplementary Table 7). Duplicated BUSCO score of the GDDH18 assembly was high, 55.0% [viridiplantae_odb12 {*n* = 822}, Table 1]. The ancestor of apple underwent a recent WGD around 27 Mya (Lallemand et al. 2023), likely explaining this high fraction of duplicated BUSCOs in the assembly.

The LAI score imputed for the assembly was 22.16, higher than the score of the GDDH13 assembly of 20.16 (Supplementary Table 7). An LAI score above 20 is characteristic of high-quality (“gold standard”) assemblies (Ou et al. 2018). Accordingly, a high LAI reflects a good continuity of repetitive genomic regions.

The short-read polishing step did not enhance the BUSCO scores and resulted in a slight drop in the LAI score (Supplementary Table 12). This could be explained by the low mean depth of the short reads available for the GDDH18 tree, around 17× after mapping and filtering of the properly paired reads. Therefore, we propose a final assembly that does not include results from the polishing step. It has recently been suggested that assemblies using Oxford Nanopore R10.4 and the latest Kit V14 technology may not require a short-read polishing step (Sereika et al. 2022; Belinchon-Moreno et al. 2025).

### Gene annotation

De novo annotation with EuGene allowed the identification of 65,170 predicted genes, of which 51,892 corresponded to protein-coding genes and 13,278 to noncoding RNA genes (Supplementary Table 13). Protein-coding genes had a mean gene length of 3,130 bp, with the shortest gene spanning 150 bp and the longest 79,620 bp (Supplementary Table 13). About 80% of protein-coding genes contain introns. The protein-coding genes comprised on average 4.79 exons (mean exon length of 315 bp) and 3.79 introns (mean intron length of 427 bp). The average coding sequence (CDS) length was 1,075 bp, while the mean 5′ and 3′ UTR lengths were 255 and 421 bp, respectively. Noncoding RNA genes including snoRNA, rRNA, tRNA, miRNA, snRNA, and other undefined ncRNA, displayed an average length of 1,074 bp.

Completeness assessment of the predicted proteome was evaluated using BUSCO v6.0.0 (viridiplantae_odb12 dataset, 822 genes), yielding completeness scores of 96.4%, with less than 0.4% missing BUSCOs. Among the 51,892 predicted proteins, 48,379 (93%) were functionally annotated by at least 1 tool (Supplementary Table 14). Specifically, 43,063 proteins were annotated by eggNOG, 46,060 by BLAST, 38,520 by InterProScan, and 40,641 by KofamScan (Supplementary Table 14). A total of 41,686 proteins were associated with at least 1 Gene Ontology (GO) term, and 42,625 with a KEGG Orthology (KO) term (Supplementary Table 14). To assess the reliability of predicted proteins, the PSAURON machine-learning model (Sommer et al. 2025) was applied, assigning a probability score for each protein based on sequence features, conservation patterns, and length distributions. Overall, 89.3% of predicted proteins exhibited a high PSAURON confidence score (higher than 0.8). Among the 4,316 unannotated proteins, 43.8% received a significant PSAURON score indicative of genuine protein-coding potential. The majority of unannotated proteins (3,019, ie 70.7%) were short, with lengths below 100 amino acids.

**Fig. 2.**
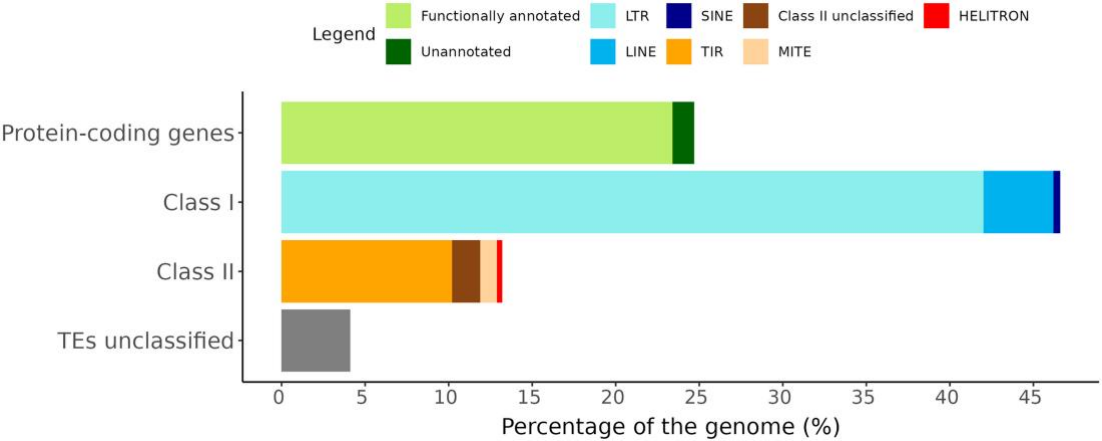
Percentage of the GDDH18 genome represented by protein-coding genes and TE regions. Percentage of the GDDH18 genome that represents the protein-coding genes and the TE regions. The class I TEs include LTR, LINE, and SINE elements. The class II TEs include TIR, unclassified class II, MITE, and HELITRON elements.

The GDDH18 assembly presented 6,239, 4,004, and 4,075 more functionally annotated protein-coding genes in comparison with GDDH13 (Daccord et al. 2017) and with the 2 haplotypes of the GDT2T genome (Su et al. 2024), respectively (Supplementary Table 7). The protein-coding and annotated genes corresponded to 23.4% of the genome (Fig. 2), in accordance with the percentage found in the GDDH13 genome (Daccord et al. 2017). A lower density of genes was observed around the putative centromeric regions of GDDH18 (Supplementary Fig. 4).

### Identification of syntenic blocks

A total of 700 syntenic blocks were identified between the chromosomes of the GDDH18 genome with i-ADHoRe (Supplementary Tables 15 to 17, Supplementary Fig. 5). These syntenic blocks were identified between chromosomes similar to GDDH13 (Lallemand et al. 2023; Supplementary Fig. 5). These syntenic blocks had a mean size of 1.5 Mb and encompassed 106 genes on average, with a median number of 31 genes. A smaller number of syntenic blocks were identified in the GDDH18 assembly in comparison with the GDDH13 assembly (865 syntenic blocks in GDDH13, Lallemand et al. 2023), and they consisted of a larger median number of genes, 31 in GDDH18, and 9 in GDDH13 (Lallemand et al. 2023), which could be further indicative that the GDDH18 assembly was less fragmented. We estimated a total of 12,006 pairs of ohnologous genes likely originating from the last WGD 27 Mya (Supplementary Table 18), in accordance with a previous study in GDDH13 (Lallemand et al. 2023).

### TE annotation

All of the 2,456 consensus had at least 1 copy on the GDDH18 genome. We found that approximately 63.2% of the GDDH18 assembly was composed of repeats, in accordance with previous results on “Golden Delicious” genomes (Daccord et al. 2017; Su et al. 2024). The class I elements were the most prevalent, representing 46.6% of the genome, including 41.9% LTR, 4.1% LINE, and 0.4% SINE (Fig. 2). DNA transposon or class II elements represented 13.2% of the genome, including 10.2% TIR, 1.0% MITE, 0.3% HELITRON, and 1.8% class II elements no further classified (Fig. 2). A total of 4.1% of copies represented unclassified TEs (Fig. 2). The proportions of class I and class II elements were similar as the GDDH13 genome (Daccord et al. 2017). *Gypsy* and *Copia* LTR elements were present in proportions close to those reported for the GDT2T genome (Su et al. 2024), representing approximately 24.8% and 12.6%, respectively.

### HODOR repeats

We found HODOR (High cOpy golDen deliciOus Repeat, accession KX869746, Daccord et al. 2017) repeats gathered on specific genomic regions of each chromosome (Supplementary Fig. 6). These genomic regions enriched in HODOR repeats correspond to the putative centromeres of the apple chromosomes (Su et al. 2024) and were found in similar regions in GDDH18 and GDDH13 (Supplementary Fig. 6). The high repetitiveness of the HODOR sequence might partially explain the fragmentation of the GDDH13 assembly obtained with shorter reads (Daccord et al. 2017).

### Inversions found between the GDDH13 and GDDH18 assemblies

Alignments of the GDDH18 and the GDDH13 chromosomes showed an overall continuity between homologous chromosomes (Fig. 1).

Some DNA sequence inversions were identified between GDDH13 and GDDH18 on chromosomes 1, 8, and 16 (Supplementary Fig. 7). These putative inversions were also found when comparing GDDH13 and GDT2T (Su et al. 2024), but they were not found when comparing GDDH18 to the 2 haplotypes of GDT2T (Supplementary Fig. 7), suggesting that sequences at these particular loci might have been misassembled in GDDH13.

Other putative inversions were found when comparing the chromosomes 2, 3, 6, and 12 of GDDH18 to one of the haplotypes of GDT2T (Supplementary Fig. 8). These putative inversions were also previously observed when comparing GDDH13 to GDT2T (Su et al. 2024). These putative inversions are close to the centromeres (Su et al. 2024). While these inverted sequences may originate from a mis-assembly, it has already been proposed and observed that centromeric regions might be naturally prone to inversions (Harringmeyer and Hoesktra 2022; Salson et al. 2025). These putative inversions have to be validated with an independent method such as Hi-C data.

## Supplementary Material

jkag104_Supplementary_Data

## Data Availability

The GDDH18 ONT long reads, the genome assembly, and the annotation of the genes are available in the European Nucleotide Archive (ENA), under the project PRJEB97937: https://www.ebi.ac.uk/ena/browser/view/PRJEB97937. The raw long reads can be found at https://www.ebi.ac.uk/ena/browser/view/ERR15761846, the assembly at https://www.ebi.ac.uk/ena/browser/view/GCA_977014495, and the annotation file with the functionally annotated genes at https://www.ebi.ac.uk/ena/browser/view/ERZ28547962. The plastid sequence, the GFF3 of all the genes annotation file, and the transposable element annotation file have been deposited via the GSA Figshare portal (https://doi.org/10.25387/g3.32012313) and can be shared upon request. The scripts used for the GDDH18 assembly and the analysis of the genome can be found on the GitLab repository: https://forge.inrae.fr/OPTIMAE/gddh18_assembly. Supplemental material available at G3 online.

## References

[jkag104-B1] Altschul SF, Gish W, Miller W, Myers EW, Lipman DJ. 1990. Basic local alignment search tool. J Mol Biol. 215:403–410. 10.1016/S0022-2836(05)80360-2. 2231712

[jkag104-B2] Aramaki T et al 2020. KofamKOALA: KEGG Ortholog assignment based on profile HMM and adaptive score threshold. Bioinformatics. 36:2251–2252. 10.1093/bioinformatics/btz859. 31742321 PMC7141845

[jkag104-B3] Astashyn A et al 2024. Rapid and sensitive detection of genome contamination at scale with FCS-GX. Genome Biol. 25:60. 10.1186/s13059-024-03198-7. 38409096 PMC10898089

[jkag104-B5] Aury JM, Istace B. 2021. Hapo-G, haplotype-aware polishing of genome assemblies with accurate reads. NAR Genom Bioinform. 3:. 10.1093/nargab/lqab034. PMC8092372 33987534

[jkag104-B4] Aury JM et al 2022. Long-read and chromosome-scale assembly of the hexaploid wheat genome achieves high resolution for research and breeding. Gigascience. 11:giac034. 10.1093/gigascience/giac034. 35482491 PMC9049114

[jkag104-B6] Balzergue S, Jeauffre J, Bahut M. 2024. Plan de gestion de données Plateau technique Analyses desAcides Nucléiques (ANAN). Université d’Angers (UA), Angers, FRA. ⟨hal-04599612v2⟩.

[jkag104-B7] Belinchon-Moreno J et al 2025. Nuclear and organelle genome assemblies of 5 *Cucumis melo* L. accessions, Ananas, Canton, PI 414723, Vedrantais, and Zhimali, belonging to diverse botanical groups. G3 (Bethesda). 15:jkaf098. 10.1093/g3journal/jkaf098. 40359376 PMC12239611

[jkag104-B8] Belser C et al 2018. Chromosome-scale assemblies of plant genomes using nanopore long reads and optical maps. Nat Plants. 4:879–887. 10.1038/s41477-018-0289-4. 30390080

[jkag104-B9] Belser C et al 2021. Telomere-to-telomere gapless chromosomes of banana using nanopore sequencing. Commun Biol. 4:1047. 10.1038/s42003-021-02559-3. 34493830 PMC8423783

[jkag104-B10] Cabanettes F, Klopp C. 2018. D-GENIES: dot plot large genomes in an interactive, efficient and simple way. PeerJ. 6:e4958. 10.7717/peerj.4958. 29888139 PMC5991294

[jkag104-B11] Camacho C et al 2009. BLAST+: architecture and applications. BMC Bioinformatics. 15:421. 10.1186/1471-2105-10-421. PMC2803857 20003500

[jkag104-B12] Cantalapiedra CP, Hernandez-Plaza A, Letunic I, Bork P, Huerta-Cepas J. 2021. eggNOG-mapper v2: functional annotation, orthology assignments, and domain prediction at the metagenomic scale. Mol Biol Evol. 38:5825–5829. 10.1093/molbev/msab293. 34597405 PMC8662613

[jkag104-B13] Carrere S, Gouzy J. 2023. Eukaryote EuGene pipeline Version 2 (2.0.0). Zenodo. 10.5281/zenodo.7648710

[jkag104-B14] Cheng H et al 2022. Haplotype-resolved assembly of diploid genomes without parental data. Nat Biotechnol. 40:1332–1335. 10.1038/s41587-022-01261-x. 35332338 PMC9464699

[jkag104-B15] Cheng H, Asri M, Lucas J, Koren S, Li H. 2024. Scalable telomere-to-telomere assembly for diploid and polyploid genomes with double graph. Nat Methods. 21:967–970. 10.1038/s41592-024-02269-8. 38730258 PMC11214949

[jkag104-B16] Cheng H, Concepcion GT, Feng X, Zhang H, Li H. 2021. Haplotype-resolved de novo assembly using phased assembly graphs with hifiasm. Nat Methods. 18:170–175. 10.1038/s41592-020-01056-5. 33526886 PMC7961889

[jkag104-B19] Cornille A, Giraud T, Smulders MJ, Roldán-Ruiz I, Gladieux P. 2014. The domestication and evolutionary ecology of apples. Trends Genet. 30:57–65. 10.1016/j.tig.2013.10.002. 24290193

[jkag104-B17] Cornille A et al 2012. New insight into the history of domesticated apple: secondary contribution of the European wild apple to the genome of cultivated varieties. PLoS Genet. 8:e1002703. 10.1371/journal.pgen.1002703. 22589740 PMC3349737

[jkag104-B18] Cornille A et al 2019. A multifaceted overview of apple tree domestication. Trends Plant Sci. 24:770–782. 10.1016/j.tplants.2019.05.007. 31296442

[jkag104-B20] Daccord N et al 2017. High-quality *de novo* assembly of the apple genome and methylome dynamics of early fruit development. Nat Genet. 49:1099–1106. 10.1038/ng.3886. 28581499

[jkag104-B21] Danecek P et al 2021. Twelve years of SAMtools and BCFtools. GigaScience. 10:giab008. 10.1093/gigascience/giab008. 33590861 PMC7931819

[jkag104-B22] Duan N et al 2017. Genome re-sequencing reveals the history of apple and supports a two-stage model for fruit enlargement. Nat Commun. 8:249. 10.1038/s41467-017-00336-7. 28811498 PMC5557836

[jkag104-B23] Flutre T, Duprat E, Feuillet C, Quesneville H. 2011. Considering transposable element diversification in de novo annotation approaches. PLoS One. 6:e16526. 10.1371/journal.pone.0016526. 21304975 PMC3031573

[jkag104-B24] Goremykin VV, Lockhart PJ, Viola R, Velasco R. 2012. The mitochondrial genome of *Malus domestica* and the import-driven hypothesis of mitochondrial genome expansion in seed plants. Plant J. 71:615–626. 10.1111/j.1365-313X.2012.05014.x. 22469001

[jkag104-B25] Harringmeyer OS, Hoekstra HE. 2022. Chromosomal inversion polymorphisms shape the genomic landscape of deer mice. Nat Ecol Evol. 6:1965–1979. 10.1038/s41559-022-01890-0. 36253543 PMC9715431

[jkag104-B26] Harris SA, Robinson JP, Juniper BE. 2002. Genetic clues to the origin of the apple. Trends Genet. 18:426–430. 10.1016/s0168-9525(02)02689-6. 12142012

[jkag104-B27] Hoede C et al 2014. PASTEC: an automatic transposable element classification tool. PLoS One. 9:e91929. 10.1371/journal.pone.0091929. 24786468 PMC4008368

[jkag104-B28] Istace B et al 2021. Sequencing and chromosome-scale assembly of plant genomes, *Brassica rapa* as a use case. Biology (Basel). 10:732. 10.3390/biology10080732. 34439964 PMC8389630

[jkag104-B29] Jones P et al 2014. InterProScan 5: genome-scale protein function classification. Bioinformatics. 30:1236–1240. 10.1093/bioinformatics/btu031. 24451626 PMC3998142

[jkag104-B30] Kim D, Song L, Breitwieser FP, Salzberg SL. 2016. Centrifuge: rapid and sensitive classification of metagenomic sequences. Genome Res. 26:1721–1729. 10.1101/gr.210641.116. 27852649 PMC5131823

[jkag104-B31] Kolmogorov M, Yuan J, Lin Y, Pevzner PA. 2019. Assembly of long, error-prone reads using repeat graphs. Nat Biotechnol. 37:540–546. 10.1038/s41587-019-0072-8. 30936562

[jkag104-B32] Kong W, Wang Y, Zhang S, Yu J, Zhang X. 2023. Recent advances in assembly of complex plant genomes. Genomics Proteomics Bioinformatics. 21:427–439. 10.1016/j.gpb.2023.04.004. 37100237 PMC10787022

[jkag104-B33] Krzywinski M et al 2009. Circos: an information aesthetic for comparative genomics. Genome Res. 19:1639–1645. 10.1101/gr.092759.109. 19541911 PMC2752132

[jkag104-B34] Lallemand T et al 2023. Insights into the evolution of ohnologous sequences and their epigenetic marks post-WGD in *Malus domestica*. Genome Biol Evol. 15:evad178. 10.1093/gbe/evad178. 37847638 PMC10601995

[jkag104-B35] Lespinasse Y, Bouvier L, Djulbic M, Chevreau E. 1998. Haploidy in apple and pear. Acta Hortic. 484:49–54. 10.17660/ActaHortic.1998.484.4.

[jkag104-B36] Li H . 2018. Minimap2: pairwise alignment for nucleotide sequences. Bioinformatics. 34:3094–3100. 10.1093/bioinformatics/bty191. 29750242 PMC6137996

[jkag104-B38] Li H, Durbin R. 2010. Fast and accurate long-read alignment with Burrows-Wheeler transform. Bioinformatics. 26:589–595. 10.1093/bioinformatics/btp698. 20080505 PMC2828108

[jkag104-B37] Li X, Ding Z, Miao H, Bao J, Tian X. 2022. Complete chloroplast genome studies of different apple varieties indicated the origin of modern cultivated apples from *Malus sieversii* and *Malus sylvestris*. PeerJ. 18:e13107. 10.7717/peerj.13107. PMC8935992 35321410

[jkag104-B39] Lin Y et al 2023. Quartet: a telomere-to-telomere toolkit for gap-free genome assembly and centromeric repeat identification. Hortic Res. 10:uhad127. 10.1093/hr/uhad127. PMC10407605 37560017

[jkag104-B40] Manni M, Berkeley MR, Seppey M, Zdobnov EM. 2021. BUSCO: assessing genomic data quality and beyond. Curr Protoc. 1:e323. 10.1002/cpz1.323. 34936221

[jkag104-B41] Ni Y, Li J, Tan Y, Shen G, Liu C. 2025. Advance in the assembly of the plant mitochondrial genomes using high-throughput DNA sequencing data of total cellular DNAs. Plant Biotechnol J. 23:4944–4965. 10.1111/pbi.70249. 40729494 PMC12576441

[jkag104-B42] Ou S, Chen J, Jiang N. 2018. Assessing genome assembly quality using the LTR assembly index (LAI). Nucleic Acids Res. 46:e126. 10.1093/nar/gky730. 30107434 PMC6265445

[jkag104-B43] Ou S, Jiang N. 2018. LTR_retriever: a highly accurate and sensitive program for identification of long terminal repeat retrotransposons. Plant Physiol. 176:1410–1422. 10.1104/pp.17.01310. 29233850 PMC5813529

[jkag104-B44] Proost S et al 2012. i-ADHoRe 3.0–fast and sensitive detection of genomic homology in extremely large data sets. Nucleic Acids Res. 40:e11. 10.1093/nar/gkr955. 22102584 PMC3258164

[jkag104-B45] Quesneville H et al 2005. Combined evidence annotation of transposable elements in genome sequences. PLoS Comput Biol. 1. 10.1371/journal.pcbi.0010022. PMC1185648 16110336

[jkag104-B46] Sallet E, Gouzy J, Schiex T. 2019. Eugene: an automated integrative gene finder for eukaryotes and prokaryotes. In: Kollmar M, editor. Gene prediction. Methods in molecular biology. Vol. 1962. Humana. p. 97–120. 10.1007/978-1-4939-9173-0_6 31020556

[jkag104-B47] Salson M et al 2023. An improved assembly of the pearl millet reference genome using Oxford Nanopore long reads and optical mapping. G3 (Bethesda). 13:jkad051. 10.1093/g3journal/jkad051. PMC10151396 36891809

[jkag104-B48] Salson M et al 2025. Interplay between large low-recombining regions and pseudo-overdominance in a plant genome. Nat Commun. 16:6458. 10.1038/s41467-025-61529-z. 40651977 PMC12255695

[jkag104-B49] Sereika M et al 2022. Oxford Nanopore R10.4 long-read sequencing enables the generation of near-finished bacterial genomes from pure cultures and metagenomes without short-read or reference polishing. Nat Methods. 19:823–826. 10.1038/s41592-022-01539-7. 35789207 PMC9262707

[jkag104-B50] Simillion C, Janssens K, Sterck L, Van de Peer Y. 2008. i-ADHoRe 2.0: an improved tool to detect degenerated genomic homology using genomic profiles. Bioinformatics. 24:127–128. 10.1093/bioinformatics/btm449. 17947255

[jkag104-B51] Sommer MJ, Zimin AV, Salzberg SL. 2025. PSAURON: a tool for assessing protein annotation across a broad range of species. NAR Genom Bioinform. 7:lqae189. 10.1093/nargab/lqae189. 39781514 PMC11704789

[jkag104-B52] Su Y et al 2024. Phased telomere-to-telomere reference genome and pangenome reveal an expansion of resistance genes during apple domestication. Plant Physiol. 195:2799–2814. 10.1093/plphys/kiae258. 38743633

[jkag104-B53] Sun X et al 2020. Phased diploid genome assemblies and pan-genomes provide insights into the genetic history of apple domestication. Nat Genet. 52:1423–1432. 10.1038/s41588-020-00723-9. 33139952 PMC7728601

[jkag104-B54] Vasimuddin M, Misra S, Li H, Aluru S. 2019. Efficient architecture-aware acceleration of BWA-MEM for multicore systems. In: *2019 IEEE International Parallel and Distributed Processing Symposium (IPDPS)*; Rio de Janeiro, Braz. IEEE. p. 314–324. 10.1109/IPDPS.2019.00041.

[jkag104-B55] Velasco R et al 2010. The genome of the domesticated apple (*Malus* × *domestica* Borkh.). Nat Genet. 42:833–839. 10.1038/ng.654. 20802477

[jkag104-B56] Zhou C et al 2025. Oatk: a de novo assembly tool for complex plant organelle genomes. Genome Biol. 26:235. 10.1186/s13059-025-03676-6. 40775726 PMC12329965

