# Supplementary material for: Assembly and annotation of the ‘Golden Delicious’ Doubled-Haploid GDDH18 apple genome
Source: G3 (Bethesda). 2026 Apr 27;16(7):jkag104. doi: 10.1093/g3journal/jkag104 (PMC13334179; doi:10.1093/g3journal/jkag104)
Supplement: jkag104_Supplementary_Data [file jkag104_supplementary_data.zip › Supplemental_Figure_Legends_G3-2026-406702.docx]

**Supplementary Figure 1.** Telomere repeats found on the extremities of the 26 contigs.

The 26 assembled contigs are presented. Violet arrows correspond to the extremities of the contigs where telomere repeats were found with quarTeT (Lin *et al.* 2023). 16 contigs present telomeric repeats at both extremities. Two contigs possess telomeric repeats at one extremity. The 17 largest contigs with two or one extremities with telomeric repeats likely correspond to the 17 apple chromosomes.

**Supplementary Figure 2.** Alignments of the contigs to the chloroplast (a) and mitochondrion (b) genome sequences.

On the left (a) is represented the alignment of the chloroplast apple sequence (NC_061549.1 NCBI accession) to the contig ptg000022c. On the right (b) is represented the alignment of the mitochondrial apple sequence (NC_018554.1 NCBI accession) to the contigs ptg000021c and ptg000024l. Dark green color corresponds to alignments with identities above 75%.

**Supplementary Figure 3.** Alignment of the plastid sequence reconstructed with the long reads to the apple chloroplast genome sequence.

Alignment of the GDDH18 plastid sequence assembled with OatK to a published chloroplast apple sequence (NC_061549.1 NCBI accession, Li et al. 2022). Dark green color corresponds to alignments with identities above 75%.

**Supplementary Figure 4.** Density of genes along the chromosomes.

The number of protein-coding and functionally annotated genes on non-overlapping genomic window of 100 kb is shown for each chromosome.

**Supplementary Figure 5.** Syntenic blocks along the chromosomes of GDDH18.

Syntenic blocks identified with i-ADHoRe are displayed. Only segments larger than 10 Mb are shown.

**Supplementary Figure 6.** Alignments of HODOR to the GDDH18 and the GDDH13 chromosomes.

Number of alignments of the 9,716 bp sequence HODOR (accession KX869746) to the GDDH18 (green line) and the GDDH13 (orange line) chromosomes. The number of HODOR alignments found on non-overlapping genomic windows of 1 Mb are shown for each chromosome.

**Supplementary Figure 7.** Alignments of the chromosomes 1, 8 and 16 of GDDH18 to both GDDH13 and the two haplotypes of GDT2T.

The chromosomes 1, 8 and 16 of the GDDH18 assembly are aligned to GDDH13 (Daccord *et al.* 2017) and to the two haplotypes of GDT2T (Su *et al.* 2024). Yellow, orange, light green and dark green colors correspond to alignments with identities between 0% and 25%, 25% and 50%, between 50% and 75%, and above 75%, respectively.

**Supplementary Figure 8.** Alignments of the chromosomes 2, 3, 6 and 12 of GDDH18 to the haplotypes of GDT2T displaying inversions.

The chromosomes 2, 3, 6 and 12 of the GDDH18 assembly are aligned to either the haplotypes 1 or 2 of GDT2T (Su *et al.* 2024). Yellow, orange, light green and dark green colors correspond to alignments with identities between 0% and 25%, 25% and 50%, between 50% and 75%, and above 75%, respectively.
