# Supplementary material for: Assembly and annotation of the ‘Golden Delicious’ Doubled-Haploid GDDH18 apple genome
Source: G3 (Bethesda). 2026 Apr 27;16(7):jkag104. doi: 10.1093/g3journal/jkag104 (PMC13334179; doi:10.1093/g3journal/jkag104)
Supplement: jkag104_Supplementary_Data [file jkag104_supplementary_data.zip › Supplementary_Tables_G3-2026-406702.docx]

**Title:** Assembly and annotation of the ‘Golden Delicious’ Doubled-Haploid GDDH18 apple genome

**Supplementary Tables**

.

**Supplementary Table 1.** Statistics of the assembly obtained with different depths.

In Excel file deposited to figshare

**Supplementary Table 2.** Transcriptome assemblies used for gene annotation.

In Excel file deposited to figshare

**Supplementary Table 3.** Raw output of Centrifuge.

In Excel file deposited to figshare

**Supplementary Table 4.** Taxonomical analysis of contaminants.

In Excel file deposited to figshare

**Supplementary Table 5.** Bacterial contamination.

In Excel file deposited to figshare

**Supplementary Table 6**. Metrics of the raw long reads and of the filtered reads used for the assembly.

| **Raw reads** |  |
| --- | --- |
| Number of bases | 82 Gb |
| Corresponding mean depth | 128X |
| Number of reads | 4,405,521 |
| Length N50 | 32 kb |
| Mean length of the reads | 19 kb |
| Number of reads > 10 kb | 2,619,334 |
| Number of reads > 30 kb | 925,346 |
| Number of reads > 50 kb | 299,665 |
| Number of reads > 100 kb | 18,120 |
| Longest read | 647 kb |
| **Filtered reads** |  |
| Number of bases | 29 Gb |
| Corresponding mean depth | 45X |
| Number of reads | 508,798 |
| Length N50 | 57 kb |
| Mean length of the reads | 57 kb |
| Longest read | 647 kb |

**Supplementary Table 7**. Comparison of the assembly statistics between GDDH13, GDDH18 and GDT2T.

|  | GDDH18 | GDDH13  (Daccord *et al*. 2017) | GDT2T hap1  (Su *et al.* 2024) | GDT2T hap2  (Su *et al.* 2024) |
| --- | --- | --- | --- | --- |
| **Contigs statistics** |  |  |  |  |
| Nbr of contigs | 28 | 2,150 | ND | ND |
| Contig N50 | 36 Mb | 620 kb | 37.7 Mb | 34.6 Mb |
| **Chromosomes statistics** |  |  |  |  |
| Total size | 655 Mb | 657 Mb | 651 Mb | 645 Mb |
| Nbr of chromosomes  with telomere repeats at both extremities | 16 | 0 | 13 | 12 |
| Nbr of chromosomes  with telomere repeats  at one extremity | 1 | 9 | 4 | 5 |
| % of N | 0 | 11.9% | 0 | 0 |
| Gaps | No gap | 3,109 | No gap | No gap |
| Chr00 total size | 2.2 Mb | 52.7 Mb | ND | ND |
| **BUSCO scores** |  |  |  |  |
| Complete BUSCOs  embryophyta (n=2,026) | 99.8% | 98.1% | 99.7% | 99.8% |
| Complete BUSCOs  viridiplantae (n=822) | 99.5% | 97.7% | 99.5% | 99.5% |
| Complete BUSCOs  rosaceae (n=10,071) | 97.4% | 96.0% | 97.3% | 97.3% |
| **LAI score** | 22.16 | 20.16 | 21.32 | 22.40 |
| Raw LAI score | 20.46 | 17.28 | 20.27 | 19.69 |
| **Protein-coding and functionally annotated genes** | 48,379 | 42,140 | 44,375 | 44,304 |

ND, no data in the publication.

**Supplementary Table 8.** Identification of telomeric repeats with quarTeT.

In Excel file deposited to figshare

**Supplementary Table 9.** OatK annotation of the chloroplastic sequence.

In Excel file deposited to figshare

**Supplementary Table 10.** Identification of contaminant sequences with FCS-GX and assignation of the contigs to the sequences of the final assembly.

In Excel file deposited to figshare

**Supplementary Table 11.**  Regions identified as adaptors and removed from the assembly.

In Excel file deposited to figshare

**Supplementary Table 12.** LAI scores before and after short-reads polishing step.

|  | LAI |
| --- | --- |
| Before polishing | 22.16 |
| After polishing | 22.05 |

**Supplementary Table 13.** Gene annotation statistics.

In Excel file deposited to figshare

**Supplementary Table 14.** Functionnal annotation results.

In Excel file deposited to figshare

**Supplementary Table 15.** Results from iADHoRe: segments.

In Excel file deposited to figshare

**Supplementary Table 16.** Results from iADHoRe: anchorpoints.

In Excel file deposited to figshare

**Supplementary Table 17.** Results from iADHoRe: multiplicon pairs.

In Excel file deposited to figshare

**Supplementary Table 18.** List of onhologous genes in GDDH18.

In Excel file deposited to figshare
