# Supplementary figures and images for: Assembly and annotation of the ‘Golden Delicious’ Doubled-Haploid GDDH18 apple genome
Source: G3 (Bethesda). 2026 Apr 27;16(7):jkag104. doi: 10.1093/g3journal/jkag104 (PMC13334179; doi:10.1093/g3journal/jkag104)

Supplementary Figure 1

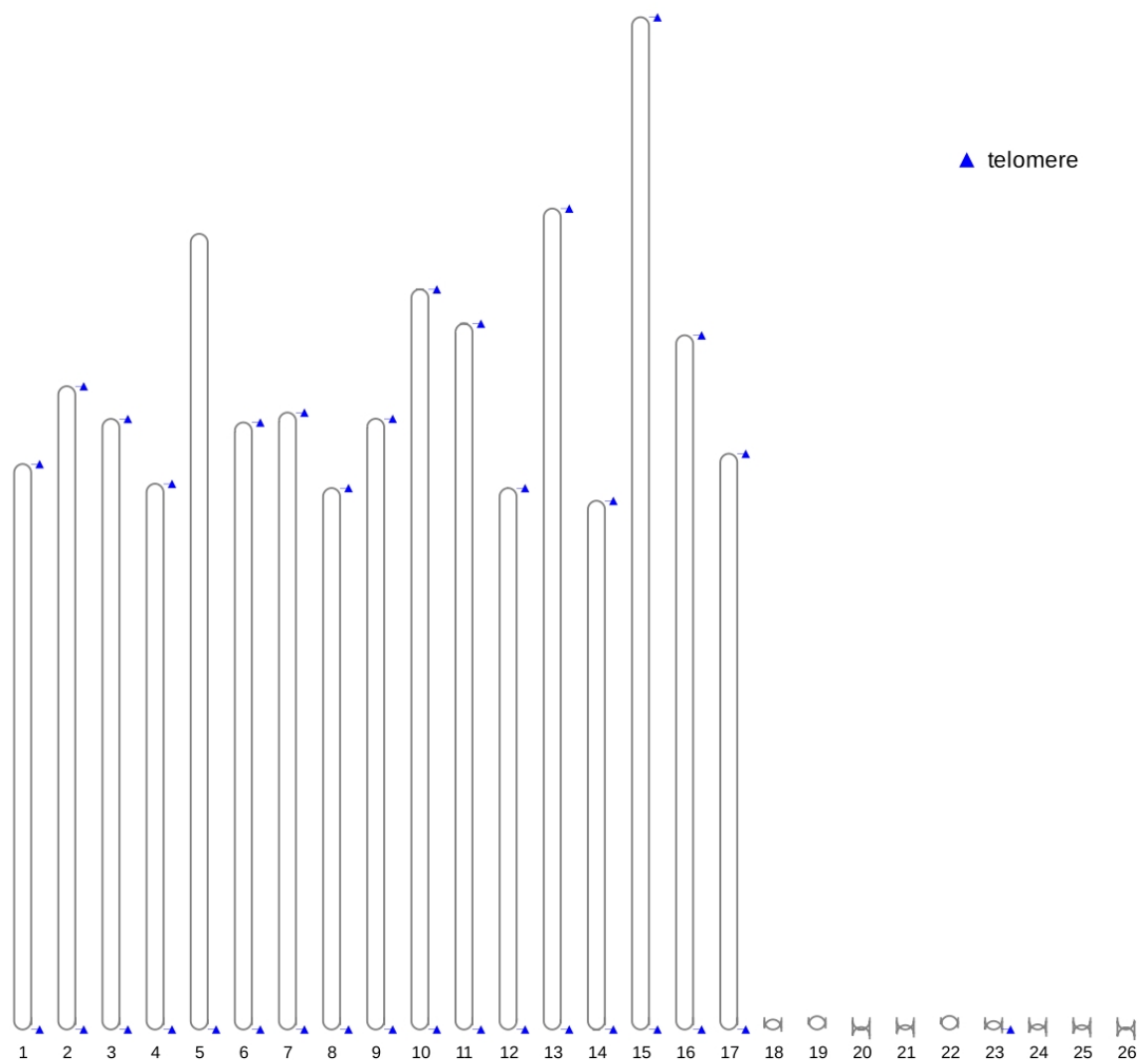

Supplement: jkag104_Supplementary_Data [file jkag104_supplementary_data.zip › Supplementary_Figure_1_G3-2026-406702.pdf]

Supplementary Figure 2

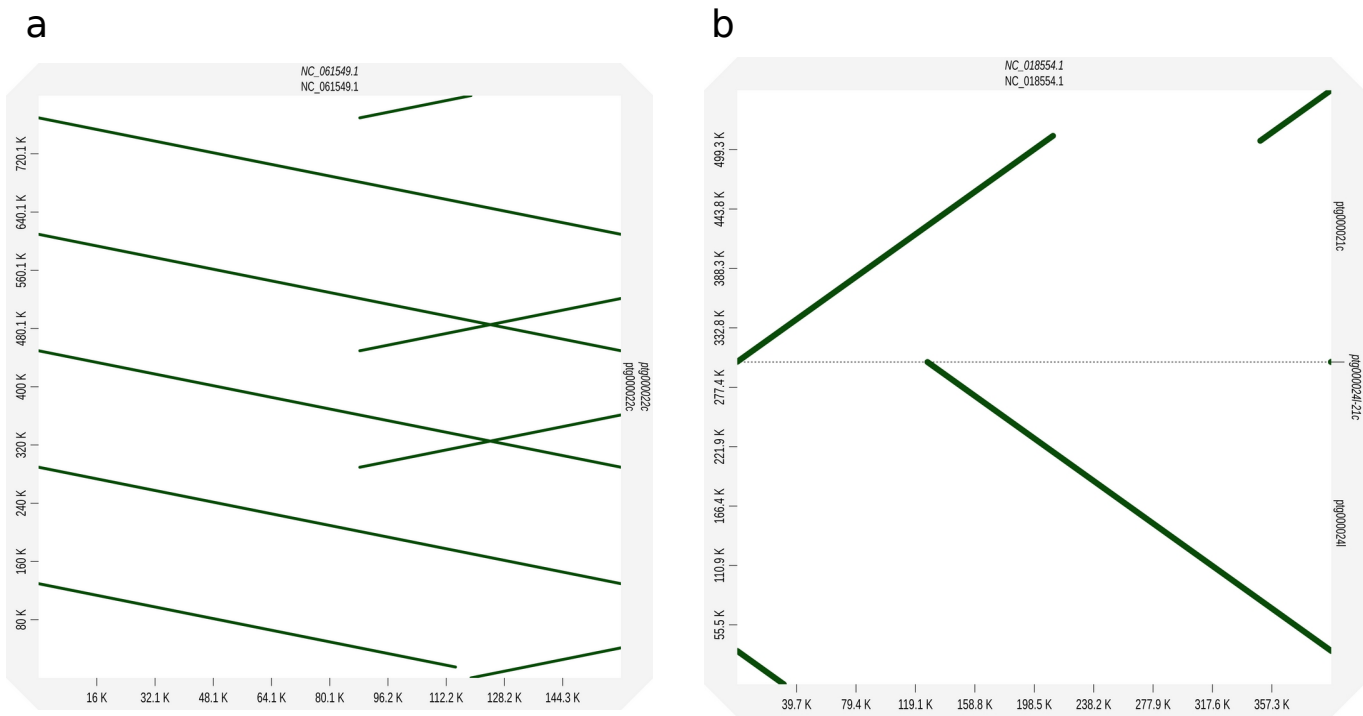

Supplement: jkag104_Supplementary_Data [file jkag104_supplementary_data.zip › Supplementary_Figure_2_G3-2026-406702.pdf]

Supplementary Figure 3

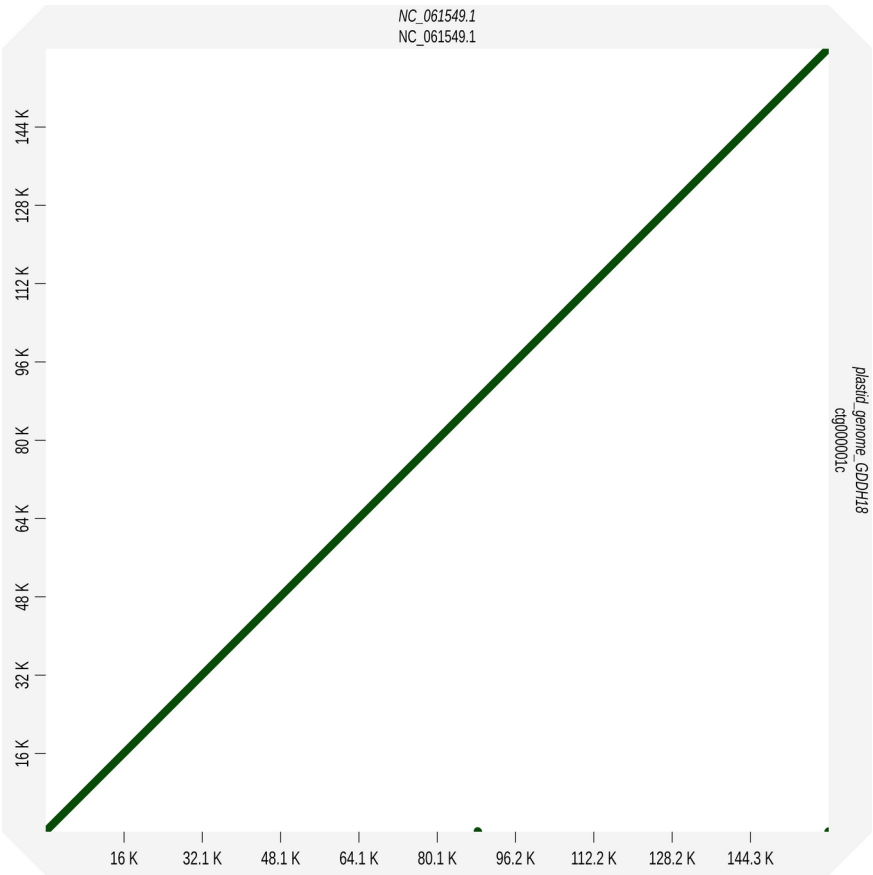

Supplement: jkag104_Supplementary_Data [file jkag104_supplementary_data.zip › Supplementary_Figure_3_G3-2026-406702.pdf]

Supplementary Figure 4

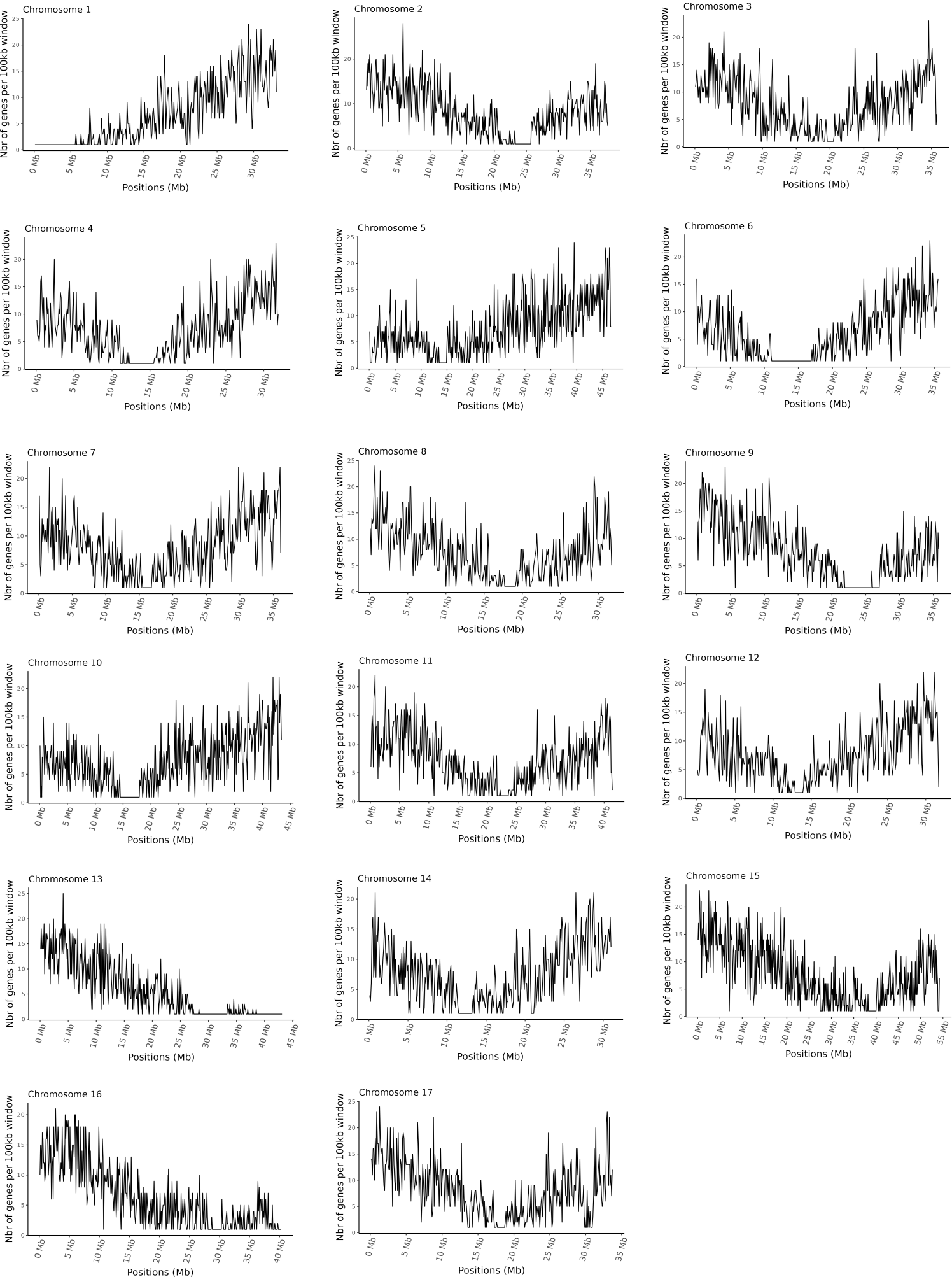

Supplement: jkag104_Supplementary_Data [file jkag104_supplementary_data.zip › Supplementary_Figure_4_G3-2026-406702.pdf]

Supplementary Figure 5

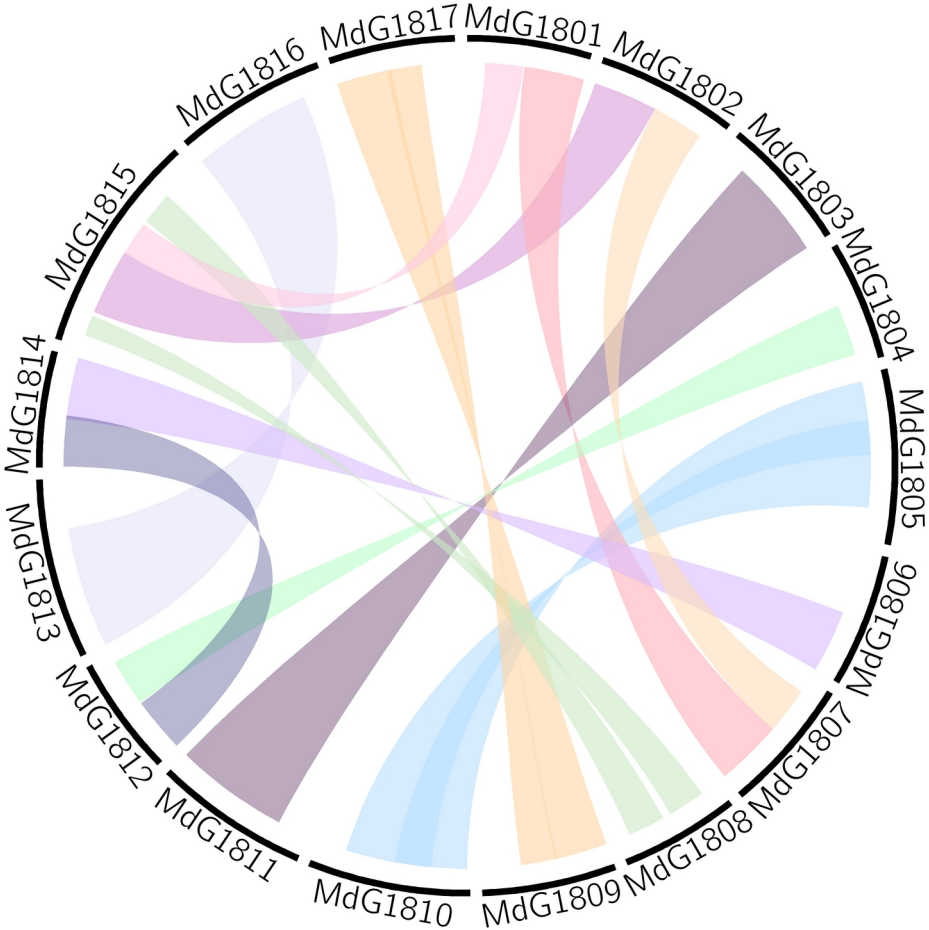

Supplement: jkag104_Supplementary_Data [file jkag104_supplementary_data.zip › Supplementary_Figure_5_G3-2026-406702.pdf]

Supplementary Figure 6

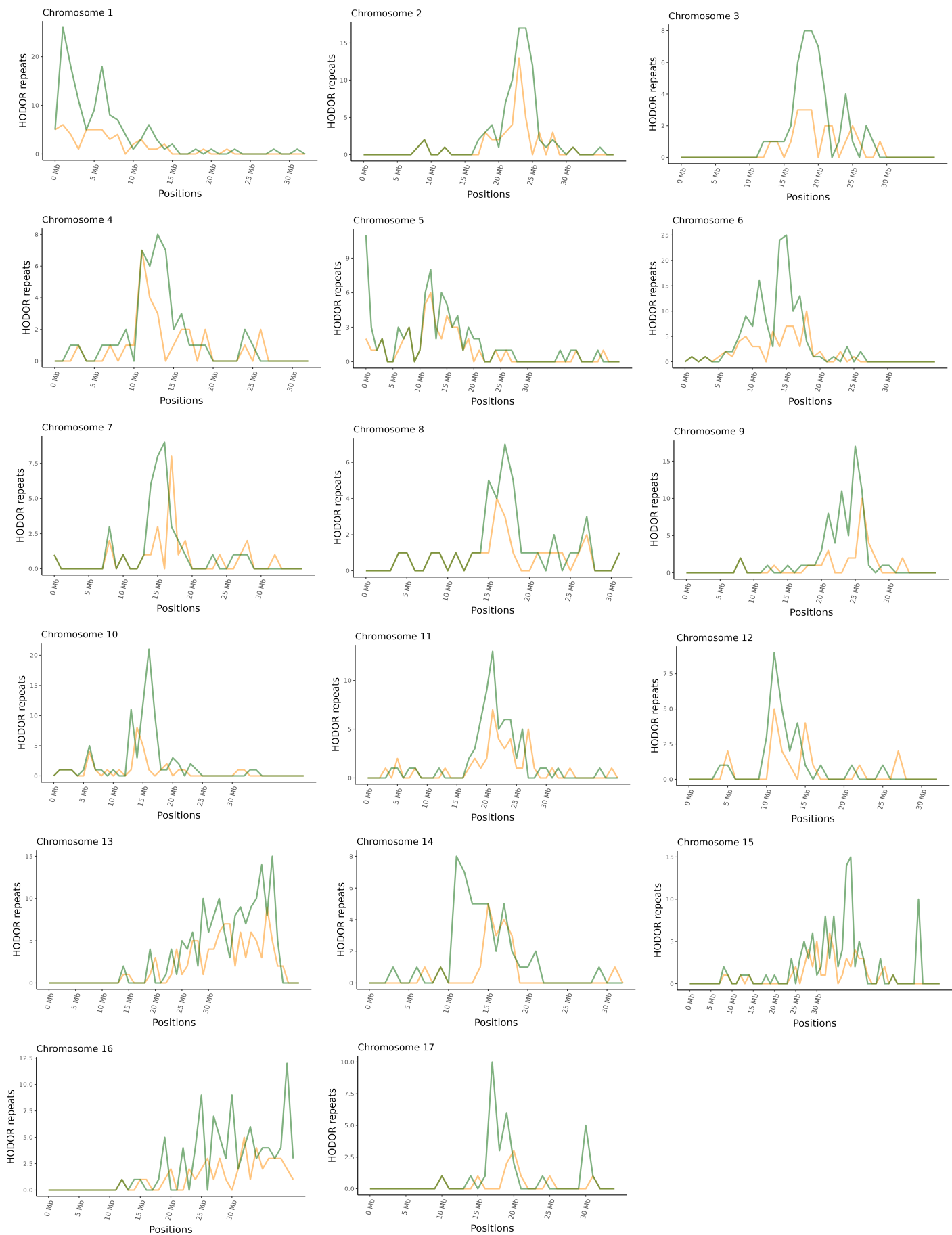

Supplement: jkag104_Supplementary_Data [file jkag104_supplementary_data.zip › Supplementary_Figure_6_G3-2026-406702.pdf]

Supplementary Figure 7

GDDH18 to GDDH13

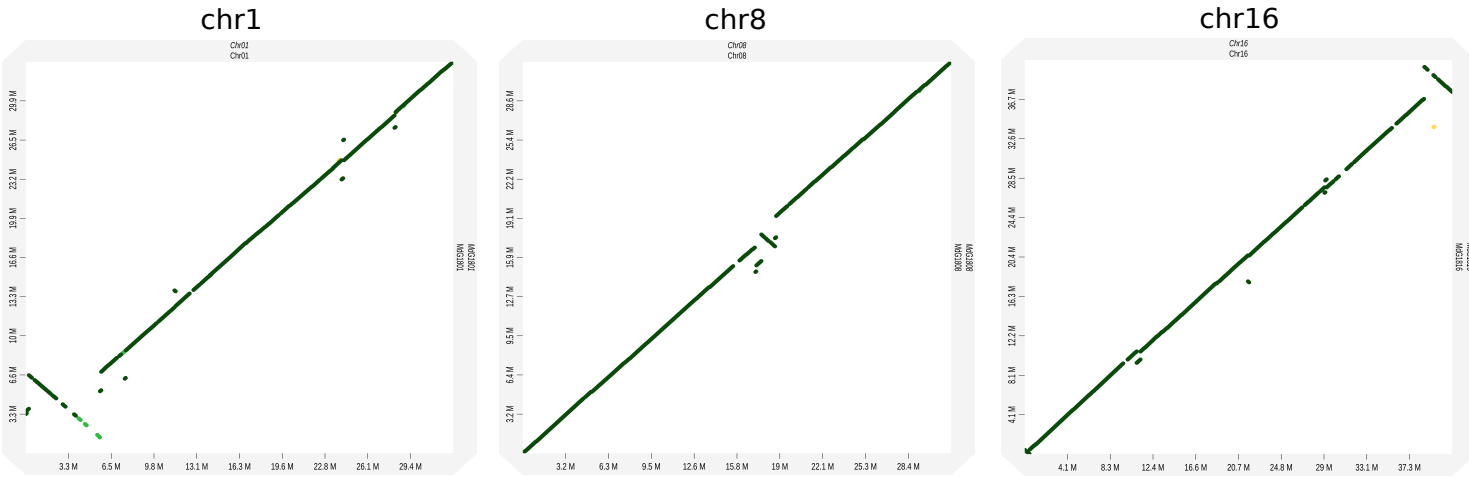

GDDH18 to GDT2T haplotype 1

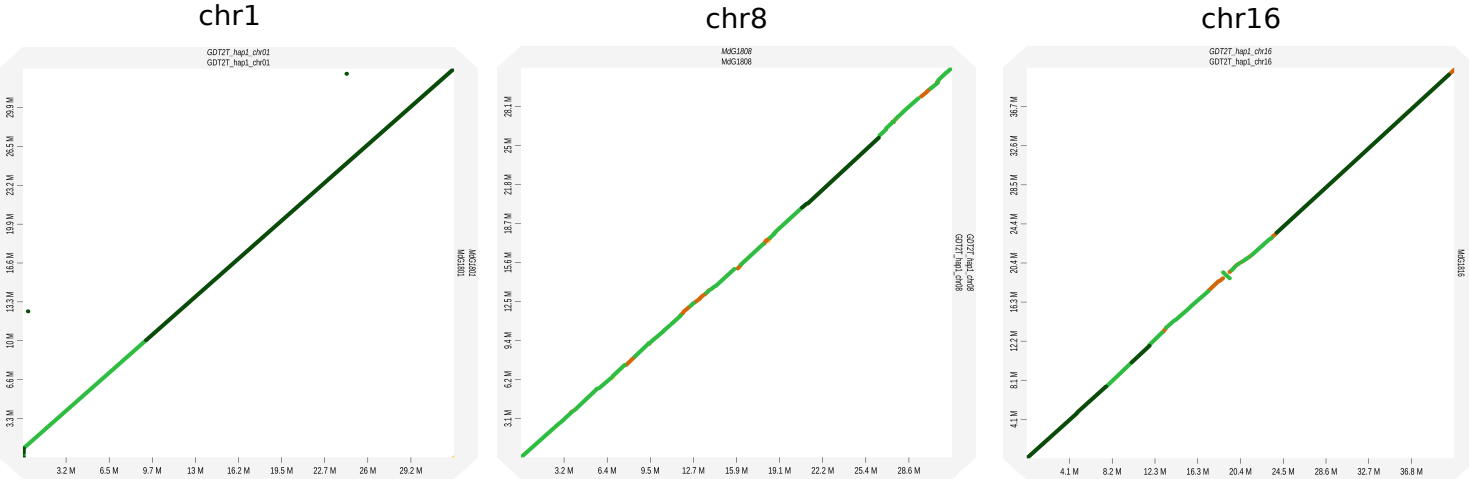

GDDH18 to GDT2T haplotype 2

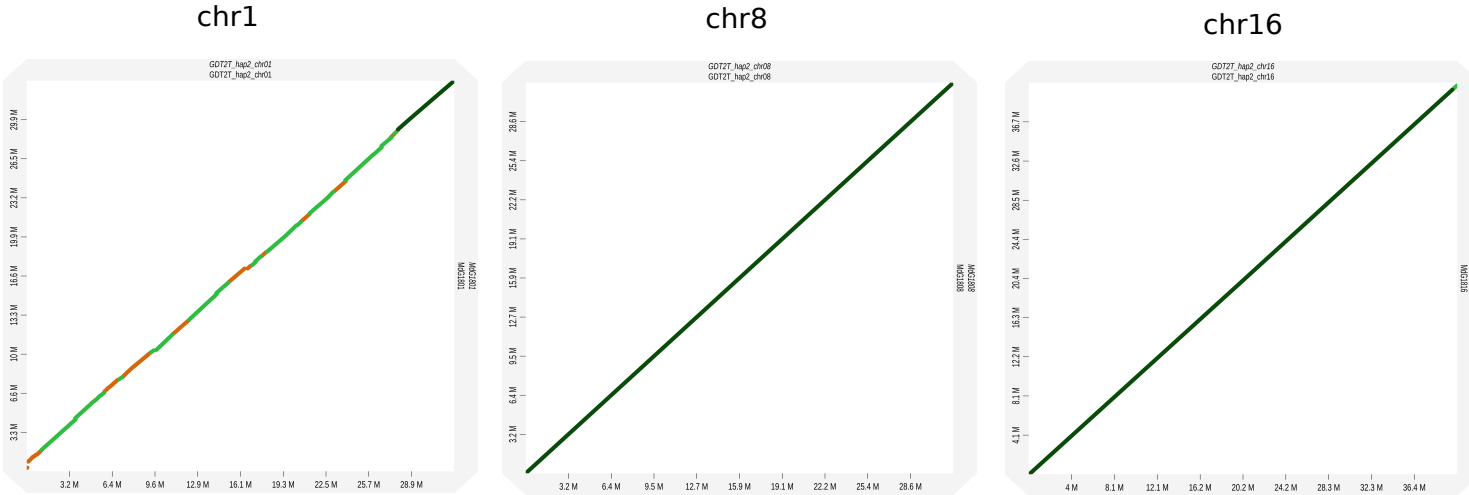

Supplement: jkag104_Supplementary_Data [file jkag104_supplementary_data.zip › Supplementary_Figure_7_G3-2026-406702.pdf]

Supplementary Figure 8

GDDH18 to GDT2T haplotype 1  
chr3

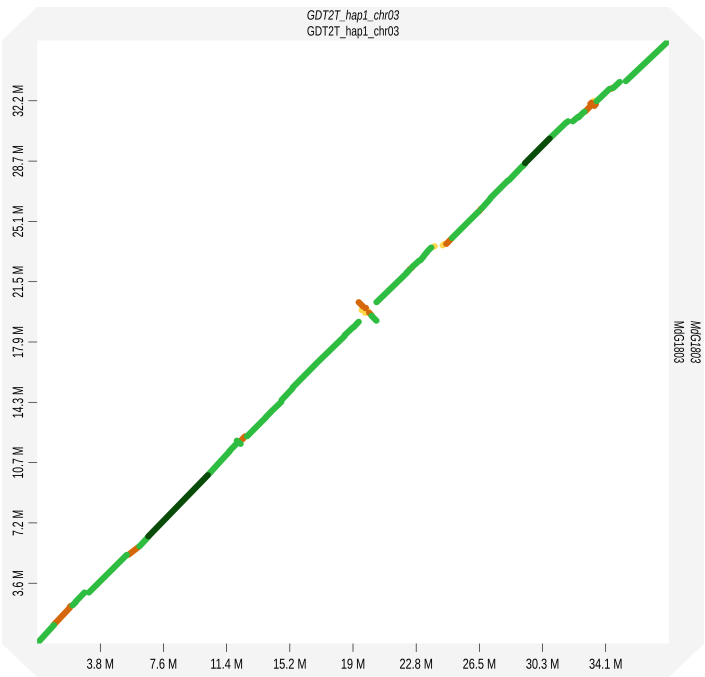

chr6

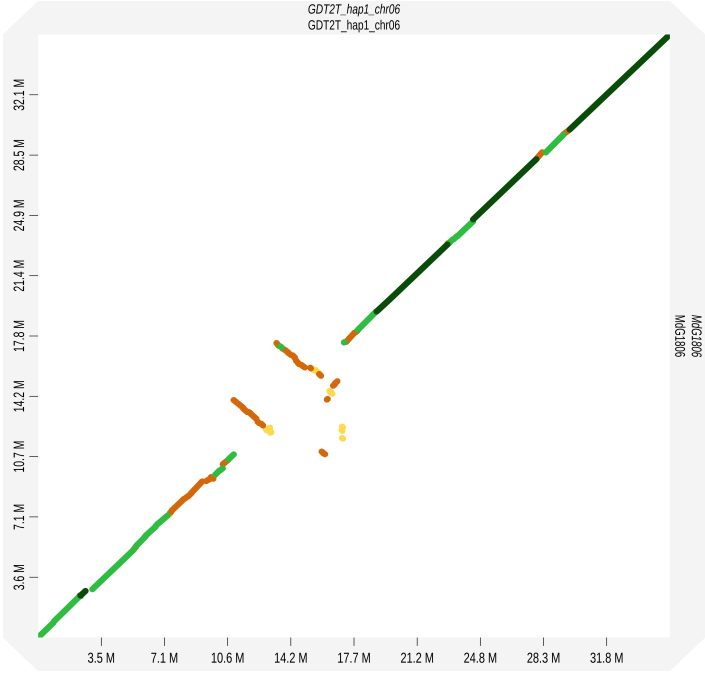

GDDH18 to GDT2T haplotype 2  
chr2

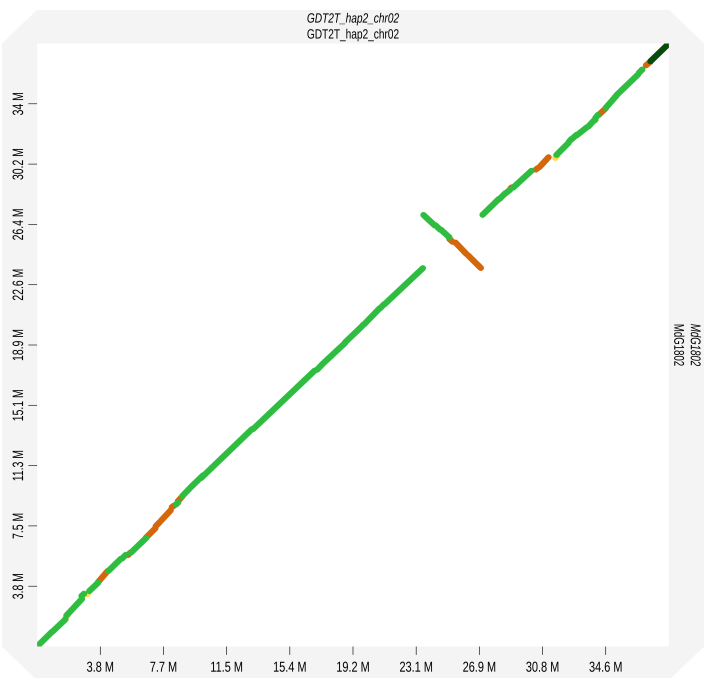

chr12

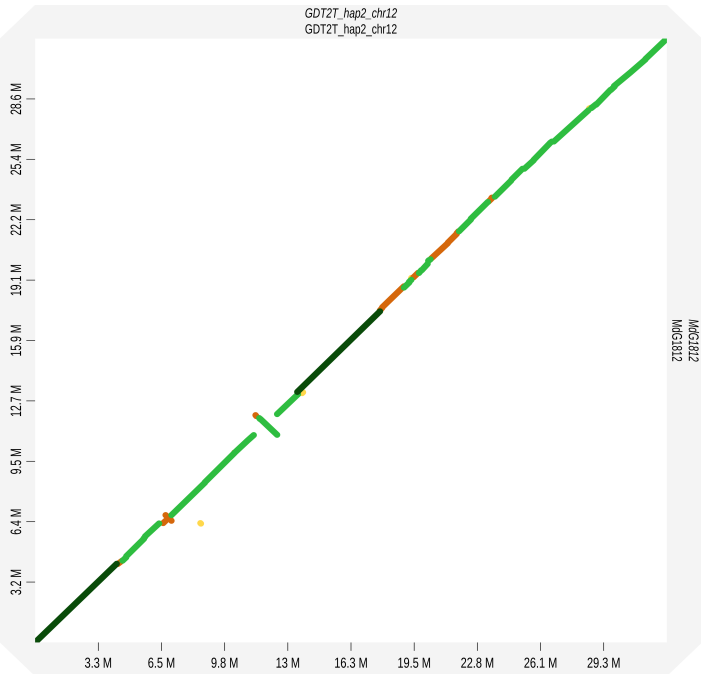

Supplement: jkag104_Supplementary_Data [file jkag104_supplementary_data.zip › Supplementary_Figure_8_G3-2026-406702.pdf]
